# Maternal exercise intervention in obese pregnancy improves the cardiovascular health of the adult male offspring

**DOI:** 10.1016/j.molmet.2018.06.009

**Published:** 2018-06-18

**Authors:** Jessica H. Beeson, Heather L. Blackmore, Sarah K. Carr, Laura Dearden, Daniella E. Duque-Guimarães, Laura C. Kusinski, Lucas C. Pantaleão, Adele G. Pinnock, Catherine E. Aiken, Dino A. Giussani, Denise S. Fernandez-Twinn, Susan E. Ozanne

**Affiliations:** 1 University of Cambridge, Metabolic Research Laboratories and MRC Metabolic Diseases Unit, Wellcome Trust-MRC Institute of Metabolic Science, Addenbrooke's Treatment Centre, Addenbrooke's Hospital, Level 4, Box 289, Hills Road, Cambridge, CB2 0QQ, UK; 2 Department of Obstetrics and Gynaecology, University of Cambridge, The Rosie Hospital and NIHR Cambridge Comprehensive Biomedical Research Centre, Box 223, Cambridge, CB2 0SW, UK; 3 Department of Physiology, Development and Neuroscience, University of Cambridge, Downing Street, CB2 3EG, UK

**Keywords:** Pregnancy, Obesity, Exercise, Hypertrophy, Echocardiography, BW, Body Weight, CVD, Cardiovascular Disease, ESV, End Systolic Volume, IVS, Interventricular Septum, LV, Left Ventricle, LVPW, Left Ventricular Posterior Wall, TD-NMR, Time Domain Nuclear Magnetic Resonance

## Abstract

**Objective:**

Obesity during pregnancy is associated with an elevated risk of cardiovascular disease in the offspring. With increased numbers of women entering pregnancy overweight or obese, there is a requirement for targeted interventions to reduce disease risk in future generations. Using an established murine model of maternal obesity during pregnancy, we investigated if a treadmill exercise intervention in the mother could improve offspring cardiac health and explored potential underlying mechanisms.

**Methods:**

A 20-minute treadmill exercise intervention protocol was performed 5 days a week in diet-induced obese female C57BL/6 mice 1 week prior to, and up to E17 of pregnancy. All male offspring were weaned onto a control diet and studied at 8 weeks of age when their cardiovascular physiology was assessed by *in vivo* echocardiography and non-invasive tail cuff plethysmography. Cardiomyocyte cell area, re-expression of fetal genes and the expression of calcium handling and sympathetic activation proteins were determined.

**Results:**

At 8 weeks, there was no difference in bodyweight or fat mass between groups. Offspring of obese dams developed pathologic cardiac hypertrophy, hypertension and cardiac dysfunction characterized by reduced ejection fraction (*p* < 0.001). Maternal exercise prevented cardiac hypertrophy and dysfunction but failed to prevent hypertension. These offspring of exercised dams also had enhanced (*p* < 0.001) levels of calcium handling proteins and a sympathetic-activated inotropic response.

**Conclusions:**

Exercise in obese pregnancy was beneficial to offspring cardiac function and structure but did not influence hypertension suggesting they are programmed by separate mechanistic pathways. These data suggest combination interventions in obese pregnancies will be required to improve all aspects of the cardiovascular health of the next generation.

## Highlights

• Maternal obesity during pregnancy causes offspring cardiovascular dysfunction.
• Maternal exercise during obese pregnancy prevented offspring cardiac dysfunction.
• Maternal exercise during obese pregnancy did not prevent offspring hypertension.
• Programming of cardiac dysfunction and hypertension occurs via divergent pathways.
• Maternal hyperinsulinemia is not required to programme offspring hypertension.

## Graphical abstract

**Figure:**
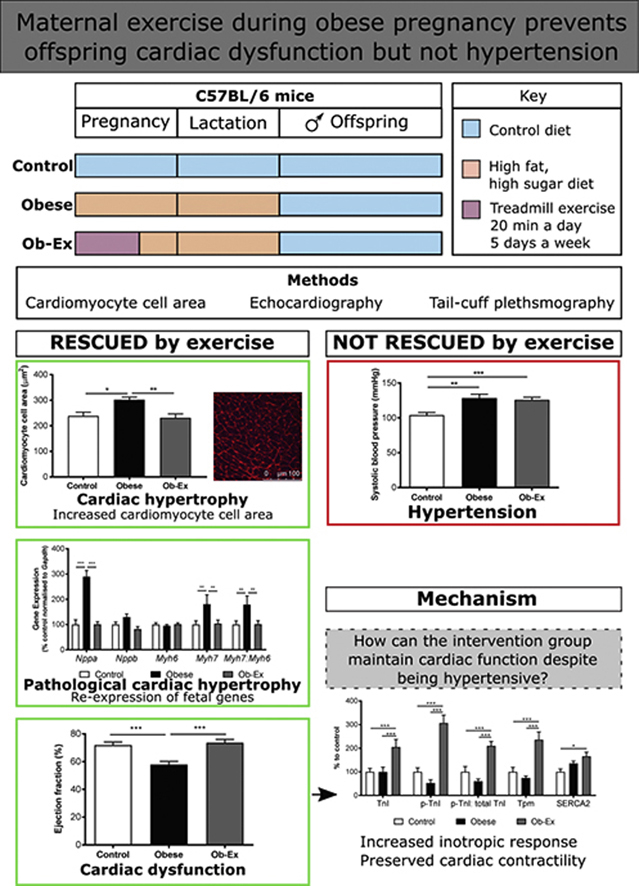

## 1 Introduction

Cardiovascular disease (CVD) is the number one cause of death globally [1]. There is already considerable understanding in the cardiovascular research field that risk factors for disease development are both modifiable (e.g. poor diet, inactivity, smoking) and non-modifiable (e.g. genetic). Prevention strategies thus far have focused specifically on attenuating disease risk by reducing an individual's modifiable risk factors postnatally. However there is considerable evidence from human and animal studies that an adverse environment *in utero* increases the risk of cardio-metabolic disease in adult life independent of genetics or their postnatal adult environment [2], [3], [4]. Therefore, there is strong rationale that disease risk could be reduced prior to birth through targeted interventions to the mother before and during pregnancy [5].

The number of women entering pregnancy either overweight or obese has risen significantly over the last decade, and in 2014 these accounted for 50% of pregnancies in the US [6]. These statistics demonstrate the scale of the problem and highlight the importance of intervention strategies not only for the immediate [7] but also the long-term health of both the mother and her child. Studies in humans have consistently shown that long-term cardiovascular health is compromised in offspring exposed to an obese pregnancy [8], [9], [10], [11], [12]. Due to rising levels of obesity worldwide, it is critical to investigate a targeted intervention achievable during an obese pregnancy to prevent transmission of poor cardiometabolic health from mother to child. Without such intervention, the burden of CVD in future generations is likely to soar. Current guidelines for exercise in pregnant women from the American Congress of Obstetricians and Gynecologists advise women with uncomplicated pregnancies to participate in at least 20–30 min of moderate aerobic and strength conditioning physical activity on most days of the week [13]. However, most women do not achieve these recommendations [14]. A number of human trials worldwide have attempted to alter lifestyle through increased physical activity and/or dietary modification [15], [16], [17], [18]. Although changing behavior is a challenge, a recent systematic review demonstrated that lifestyle (diet and physical activity) intervention strategies in pregnancy were enough to limit gestational weight gain irrespective of the mother's BMI, ethnicity and age [19]. Most human pregnancy intervention studies report only immediate effects during pregnancy and at the point of delivery. Recent findings from UPBEAT have shown that increased physical activity and a reduction in glycemic load during pregnancy, reduced offspring adiposity at 6 months of age [20]. However, due to the long-term nature of CVD and relative infancy of such intervention studies, data on such outcomes in humans will not be known for many years. There is, therefore, a need to model intervention strategies in animals to longitudinally determine the cardiometabolic effects on the offspring.

Our laboratory uses a well-established murine model of maternal diet-induced obesity in which we mimic human obesity by feeding murine dams a Western-style diet that is high in sugar and fat. Offspring develop pathologic cardiac hypertrophy [21] and importantly, the hearts of these offspring demonstrate impaired function *in vivo* and *ex vivo*, with evidence of sympathetic dominance [22], [23] as well as hypertension [24]. The aim of the current study was to use this model to test the effectiveness of exercise intervention during obese pregnancy at preventing programmed cardiovascular dysfunction in the adult offspring and to identify underlying molecular mechanisms.

## 2 Methods

### 2.1 Animals

Studies were conducted according to the UK Home Office Animals (Scientific Procedures) Act 1986 and following ethical review and approval by the University of Cambridge Animal Welfare and Ethical Review Board. This study capitalized on an already established maternal diet-induced obesity model using C57BL/6J mice [25]. At 3 weeks of age, female mice were randomly assigned to receive either a standard laboratory RM1 diet (approx. 7% simple sugars, 3% fat, 50% polysaccharide, 15% protein [w/w], 10.74 kJ/g)] or a semi-synthetic, energy-rich, highly palatable obesogenic diet (approx. 10% simple sugars, 20% animal lard, 28% polysaccharide, 23% protein [w/w], 28.43 kJ/g) supplemented with sweetened condensed milk (Nestle, UK) (approx. 16% fat, 33% simple sugars, 15% protein, 13.7 kJ/g), which was fortified with mineral and vitamin mix AIN93G. Both diets were purchased from Special Dietary Services (UK). Detailed energy content is supplied in Supplementary Table 1. A first pregnancy is undertaken to prove fertility and establish good maternal care. The interval between the weaning of the first pregnancy and mating for the second pregnancy was at least one week and animals were maintained on the same diet throughout both pregnancies. From this point, body composition was assessed using Time Domain-Nuclear Magnetic Resonance (TD-NMR); this method was chosen to allow longitudinal non-invasive measurement of body composition in the dams both before and during pregnancy in the same animal. Once females on the obesogenic diet reached an absolute fat mass greater than 10 g, they were considered obese and were mated for the second pregnancy. Control females were only entered into the study if they had an absolute fat mass under 5 g. These 2 groups of females were rested for one week before mating (to match for the training week undertaken by the obese-exercised group), and weekly TD-NMR measurements were taken before mating and throughout pregnancy. Females were mated with previously LAD1 fed males (diets were matched to the incoming female during the period of mating) and maintained on their respective diets throughout pregnancy and lactation. At the time females were mated for their second pregnancy, they had been on the diet for approximately 15 weeks and were approximately 18 weeks old.

### 2.2 Maternal exercise intervention

For the third group, obese females (>= 10 g fat mass) were trained to run on a treadmill. Treadmill speed was gradually increased over 5 days of training. By day 5, mice were running 20 min per day at a top speed of 12.5 m/min. Mice were time mated after 1 week of training. A 5-day exercise protocol (weekdays) followed by 2 days rest (weekend) was continued up to and including gestational day 17. These mice are referred to as Obese-Exercise (Ob-Ex). The experimental protocol is shown schematically as part of the graphical abstract.

Litter size (all groups) was standardized to 6 pups on postnatal day 2, and only male offspring were studied to avoid the influence of sex. At weaning, all offspring were weaned onto RM1. The N for all experiments corresponds to the number of independent litters (number of dams, Control n = 16, Obese n = 16 and Ob-Ex n = 7). To eliminate confounding within litter effects, only one male per litter was used for each outcome measure. TD-NMR measurements were undertaken weekly in male offspring from 4 to 8 weeks of age. At eight weeks of age, mice were fasted overnight (16 h). Tail blood glucose was measured (Alphatrak 2, Zoetis, USA) before the mice were killed by rising CO_2_. Immediately *postmortem*, blood was collected by cardiac puncture and tissue was isolated. Our study aimed to address if exercise during an obese pregnancy was effective at reducing the long-term detrimental cardiovascular effects on the offspring. As we were not seeking to investigate the impact of exercise during a lean pregnancy, we did not include an exercised control group of dams. This was to ensure that we were in keeping with the ARRIVE guidelines (NC3Rs) and only utilized animals required to fulfil our specific research aims.

### 2.3 Serum analysis

Total serum cholesterol, free fatty acids and triglycerides were measured by the MRC MDU Mouse Biochemistry Laboratory (Addenbrooke's Hospital, UK).

### 2.4 Histology

Hearts were immersion-fixed in 10% neutral buffered formalin, processed and sectioned at 10 μm. Using a previously published method [23], three mid-cardiac sections from each heart were stained; six hearts (from independent litters) were analyzed per experimental group. Wheat germ agglutinin [TexasRed-X conjugate, (Molecular Probes, Life Technologies, USA)] was used to stain cardiomyocyte cell borders. Images were analyzed double-blinded using CellD software (Olympus, Japan). Only myocytes in cross-section in which cell borders were clearly visible were measured. Mid-cardiac cardiomyocyte cell area was calculated by averaging across at least three fields of view randomly captured across the LV in the mid-cardiac section and three mid-cardiac sections were analyzed per heart (∼7000 cells were counted/experimental group).

### 2.5 Quantitative PCR

Snap-frozen hearts were powdered on dry ice. RNA was extracted using a miRNeasy mini kit (Qiagen, Germany) following the manufacturers protocol. The reverse transcriptase reaction was performed using a high capacity cDNA RT kit (ABI, USA). Real time qPCR was carried out in a 384 well plate using SYBR green PCR master mix (ABI, USA) and performed using a 7900HT fast real-time PCR system (ABI). All data were normalized to the expression of a housekeeping gene, *Gapdh*- Glyceraldehyde 3-phosphate dehydrogenase, the expression of which was not different between groups (Control, 100 ± 3.1%; Obese, 99.1 ± 4.5%; Ob-Ex, 100.4 ± 4.6%). Gene expression was measured for *Nppa*- atrial natriuretic peptide; *Nppb*- brain natriuretic peptide; *Myh7*- β-MHC; *Myh6*- α-MHC. Primer sequences are shown in Supplementary Table 2.

### 2.6 Measuring systolic blood pressure

Non-invasive systolic blood pressure measurements were carried out using restraint tail cuff plethysmography [BP-2000 Series II (Visitech systems, Canada)]. Mice were trained on two consecutive days prior to measurement of systolic blood pressure and pulse rate. Over the training days, the variation between measurements decreased so that by the 3^rd^ day the co-efficient of variance was less than 10%. All sessions were performed at the same time each day (1600 h) to control for the natural circadian variation of blood pressure and by the same experimenter. Tail cuff was chosen over telemetry for technical and scientific reasons. Firstly, telemetry is recommended in mice heavier than 25 g [26], [27], whereas our mice only reached this weight by *post mortem*, which was 1 week after blood pressure measurements were performed. Secondly, as echocardiography and blood pressure was performed on the same mouse, we wanted to avoid the animal having to be anaesthetized on more than one occasion.

### 2.7 Echocardiography

Left ventricular function was assessed by transthoracic echocardiography (FUJIFILM VisualSonics, Canada, Vevo 770 with a RM707B 30 MHz cardiac transducer). Anesthesia was induced via inhalation of 2% isoflurane in O_2_ and maintained at 1.5% isoflurane throughout. Mice were placed in a supine position on a heated platform to enable real time monitoring of ECG, and body temperature was taken via a rectal probe. Heart rate was constantly monitored and kept >400 beats per minute (bpm) by modulating flow of isoflurane where necessary. The transducer was carefully positioned to obtain a parasternal long-axis view of the left ventricle (LV). In B-mode the probe was adjusted to optimally align the aortic valve and apex of the heart, with the papillary muscle in view. M-mode images were captured from this position. Measurements were performed using Vevo 770 software. Cardiac functional outcomes were calculated using the LV tracing function and lumen and wall dimensions were measured using the in-built measurement platform on M-mode images. Ascending aortic diameter was measured from captured EKV™ files and the width from wall to wall at mid-systole and diastole was measured within 1 mm of the aortic valve (Supplementary Fig. 1).

### 2.8 Western blotting

Ventricular tissue was mechanically homogenized (TissueRuptor, Qiagen, Manchester, UK) in lysis buffer [50 mmol/L HEPES, pH 8; 150 mmol/L sodium chloride; 1% Triton X100; 1 mmol/L sodium orthovanadate; 30 mmol/L sodium fluoride; 10 mmol/L sodium pyrophosphate; 10 mmol/L EDTA with a protease inhibitor cocktail (set III), Calbiochem Novabiochem Biosciences, UK]. Homogenates were centrifuged, the pellet was discarded, and the supernatant placed in a fresh tube. Total protein concentration in the cleared lysate was measured using a copper/bicinchoninic assay (Sigma–Aldrich, UK). Homogenates (10 μg of protein/sample) from all 3 groups were subjected to SDS-PAGE and transferred onto a PVDF membrane (Immobilon-P, Millipore, MA, USA). Membranes were blocked with 5% skimmed milk protein (Marvel, Premier Foods, UK) in 1x Tris-buffered Saline (TBS)-0.1% Tween 20 after which they were incubated in primary antibody at 4 °C overnight. All primary antibodies were used at 1:1000 dilution and were obtained from Cell Signaling Technology (MA, USA): Tropomyosin (#3990S rabbit monoclonal), Troponin I (#4002S rabbit polyclonal), phosphorylated (Ser23/24) Troponin I (p-Troponin I) (#4004S rabbit polyclonal) and ATP2A2/SERCA2 (#4388S rabbit polyclonal). The following day they were washed in 1x TBS - 0.1% Tween 20 before incubation with horseradish peroxidase-conjugated anti-rabbit secondary antibody (Jackson ImmunoResearch, Stratech, UK). Antibody binding was detected using Super Signal West Pico Chemiluminescent substrate (Thermo Scientific, UK) and chemiluminescent bands were quantified directly with Bio-Rad ChemiDoc Imager (USA).

### 2.9 Statistical analysis

Morphometric and physiologic data were analyzed using Prism7 (Graphpad, USA). Values are expressed as mean ± SEM. For maternal data, two-way ANOVA was carried out with repeated measures to determine the overall effect of maternal group and time, over mating and gestation. Otherwise, one-way ANOVA was used to compare the three experimental groups; a Bonferroni post-hoc test was carried out when the overall *P* value for ANOVA was less than 0.05.

Cell area data were analyzed using a hierarchical linear model. A hierarchical linear model with random effects for individual animal, and an interactive effect between individual and section of origin was used to analyze cardiomyocyte cell area. The model included maternal diet and maternal exercise as fixed effects. This structure accounted for the fact that multiple cell area measurements were obtained from multiple sections of the heart of each individual animal within the groups, and these data cannot be treated as fully independent [R statistical software package version 2.14.1 (R Foundation for Statistical Computing, Austria)].

## 3 Results

### 3.1 Maternal body composition

**Figure 1:**
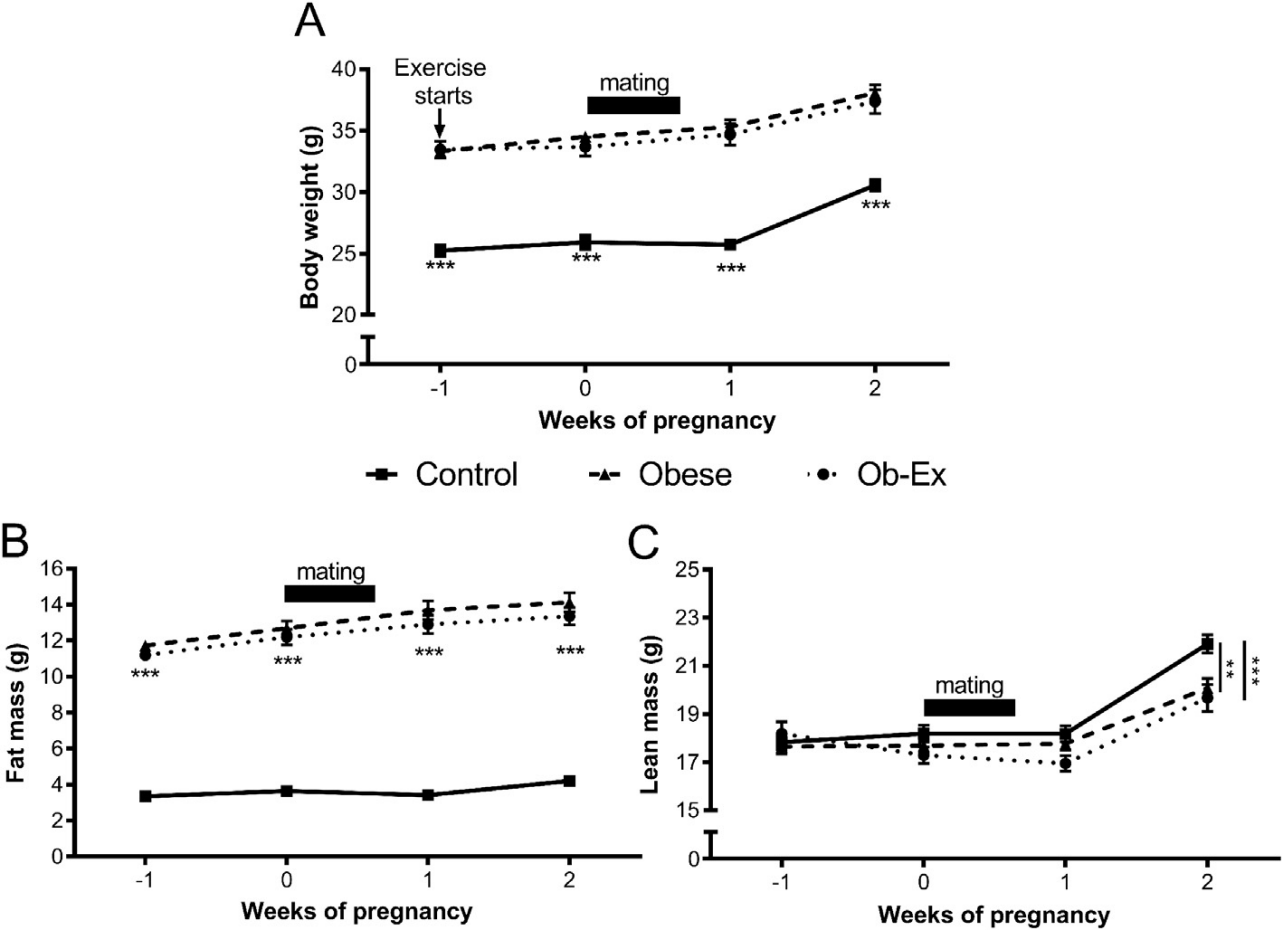
**Maternal body composition**. A) Body weight. TD-NMR was used to assess B) Fat mass and C) Lean mass of dams. Week 0 = day females were set up for timed mating. Data points are separated by 1 week. Control n = 12, Obese n = 12 and Ob-Ex n = 11. **p < 0.01 and ***p < 0.001).

Compared to controls, obese females had increased body weight at the start of pregnancy that was maintained throughout pregnancy and lactation (Figure 1A). This increase was a result of increased fat mass at all time points (Figure 1B). Maternal exercise in the obese dams did not alter maternal body weight or composition when compared to non-exercised obese dams (Figure 1).

During the second week of pregnancy, obese dams had decreased lean mass when compared to control dams. This reduction in lean mass was not ameliorated by the exercise intervention (Figure 1C). As TD-NMR measurements cannot distinguish between the body composition of the dam and her pups, this deficit could be accounted for by the fact that both Obese and Ob-Ex groups had a reduced litter size at birth (Control, 8.3 ± 0.4; Obese, 6.3 ± 0.4; Ob-Ex, 6.0 ± 0.5; *p* = 0.002) and a decreased total litter weight (Control, 11.1 ± 0.4 g; Obese, 9.6 ± 0.4 g; Ob-Ex, 8.8 ± 0.5 g; *p* = 0.003).

### 3.2 Offspring body composition

There was no difference in body weight or lean mass between the three offspring groups at 8 weeks of age. However, there was an overall trend (*p* = 0.06) for the offspring of obese dams to have increased fat mass (Table 1). Serological analysis showed no differences between offspring groups for free fatty acid and triglyceride levels. Total cholesterol was not significantly altered between offspring of control or obese dams; however the intervention group had decreased serum cholesterol when compared to control offspring (Table 2). There were no changes in fasting plasma glucose between groups. However, it has been previously reported that hyperinsulinemia in the offspring of obese dams is corrected by maternal exercise [25].

**Table 1:** Offspring body composition and heart weight.

	Control	N	Obese	N	Ob-Ex	N	p value
Fed BW (g)	25.5 ± 0.3	6	25.6 ± 0.3	6	25.6 ± 0.5	5	0.98
Fat mass (g)	2.3 ± 0.1	6	3.1 ± 0.3	6	2.6 ± 0.3	5	0.06
Lean mass (g)	18.5 ± 0.3	6	18.1 ± 0.3	6	18.1 ± 0.3	5	0.56
Fasted BW (g)	22.8 ± 0.4	11	22.4 ± 0.7	9	23.2 ± 0.3	9	0.55
Heart weight (mg)	130.6 ± 3.8	11	156.8 ± 5.7^ac^	9	135.9 ± 2.0	9	<**0.001**
Relative heart weight (%BW)	0.57 ± 0.01	11	0.66 ± 0.02^ac^	9	0.59 ± 0.01	9	<**0.001**

Body composition was performed by TD-NMR under fed conditions. 16 h fasted body weight (BW) was measured to allow normalization of fasted heart weight. *P* values were calculated by one-way ANOVA and shown in **bold** when a significant difference was detected (*p* < 0.05). Letters represent differences between specified groups by Bonferroni post-hoc test (*p* < 0.05) ^a^ Control *vs*. Obese and ^c^ Obese *vs*. Ob-Ex.

**Table 2:** Serological analysis in 8 week offspring.

Analytes (mmol/L)	Control	N	Obese	N	Ob-Ex	N	p value
Triglycerides	1.19 ± 0.07	9	1.25 ± 0.09	6	1.48 ± 0.14	9	0.14
Free Fatty Acids	926.9 ± 49.0	9	935.0 ± 105.7	6	799.6 ± 87.8	9	0.79
Total Cholesterol	3.53 ± 0.08	8	3.38 ± 0.06	5	3.18 ± 0.09^b^	9	**0.02**
Plasma Glucose	5.4 ± 0.3	9	6.1 ± 0.2	6	6.4 ± 0.4	9	0.20

Analytes were analyzed in overnight fasted (16 h) serum from male offspring at 8 weeks of age. *P* values were calculated by one-way ANOVA and shown in **bold** when a significant difference was detected (*p* < 0.05). Letters represent differences between specified groups by Bonferroni post-hoc test (*p* < 0.05) ^b^ Control *vs*. Ob-Ex.

### 3.3 Assessing cardiac hypertrophy

**Figure 2:**
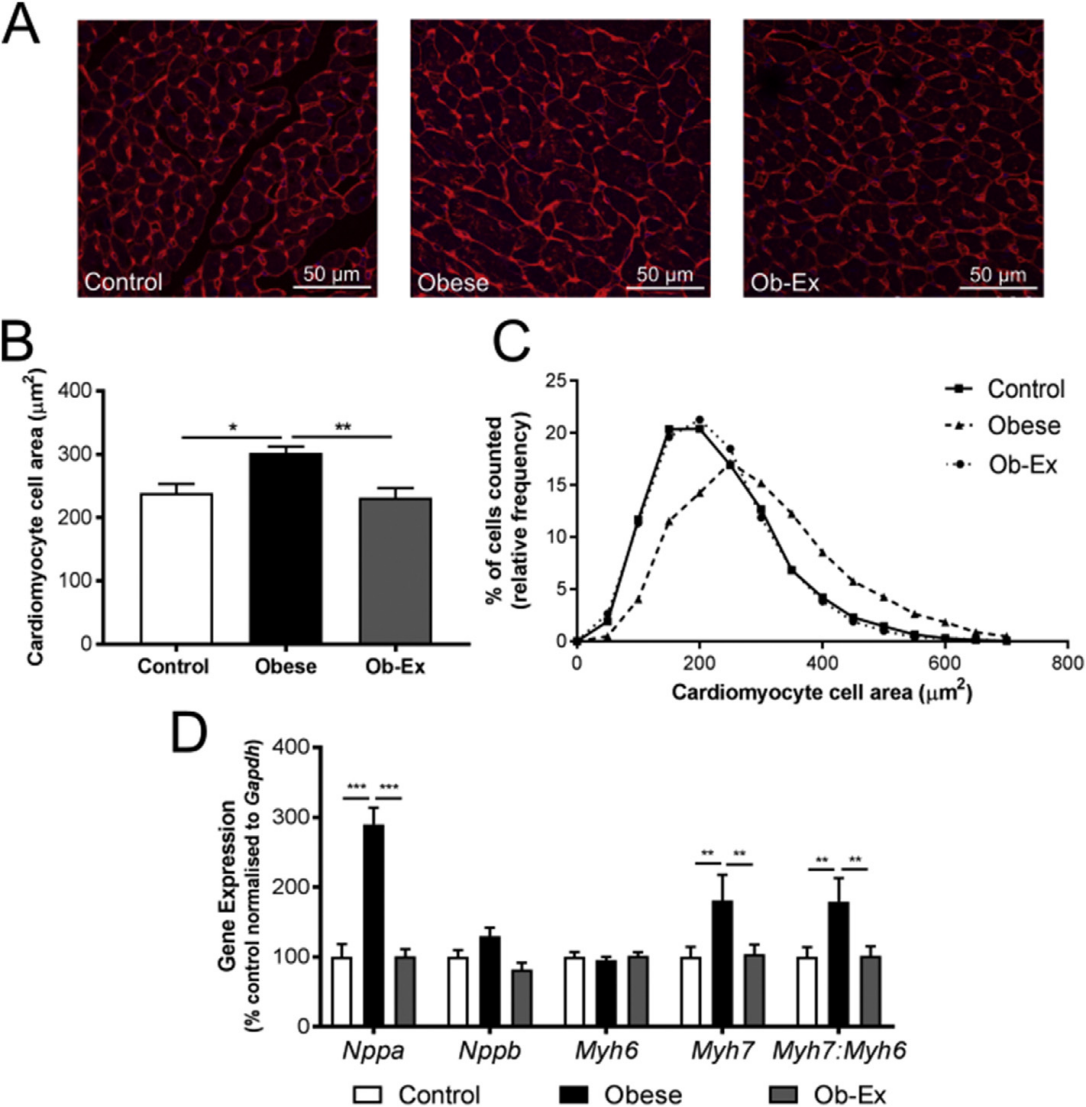
**Pathologic LV cardiac hypertrophy in male offspring at 8 weeks of age**. A) Representative images of wheat germ agglutinin stained mid-cardiac sections. B) Mean cardiomyocyte cell area. C) Frequency distribution of cardiomyocyte cell area. N = 6 hearts per group with ∼7000 cells analyzed per group. D) Ventricular fetal gene expression. Housekeeper glyceraldehyde 3-phosphate dehydrogenase (*Gapdh)* was unchanged between groups. Control n = 9, Obese n = 7 and Ob-Ex n = 9. *p < 0.05, **p < 0.01 and ***p < 0.001. *Nppa*- atrial natriuretic peptide; *Nppb*- brain natriuretic peptide; *Myh7*- β-MHC; *Myh6*- α-MHC.

Male offspring from obese dams had increased heart weight at 8 weeks of age, both in absolute terms and when expressed relative to body weight (Table 1). Cardiomyocyte cell area was also significantly increased (Figure 2A,B). These parameters were normalized in offspring from the Ob-Ex group (Table 1 and Figure 2A,B). Frequency distribution of cardiomyocyte cell areas from all groups showed a rightward shift, indicative of a higher proportion of larger cells in the offspring of obese dams, thereby confirming hypertrophy (Figure 2C). Hierarchical linear model analysis showed that the *p* value for the effect of maternal diet to increase cell size was *p* < 0.001, and for exercise to prevent this increase was *p* < 0.001.

Atrial natriuretic peptide (*Nppa)* expression was increased approximately three-fold in the obese dam offspring hypertrophic hearts; this expression was normalized in the Ob-Ex group (Figure 2D). Furthermore the *Myh7*: *Myh6* ratio was increased in the hearts of offspring exposed to maternal obesity, which was driven by increased *Myh7* rather than *Myh6* (Figure 2D). This was also normalized in the intervention group (Figure 2D).

### 3.4 Quantification of systolic blood pressure

**Figure 3:**
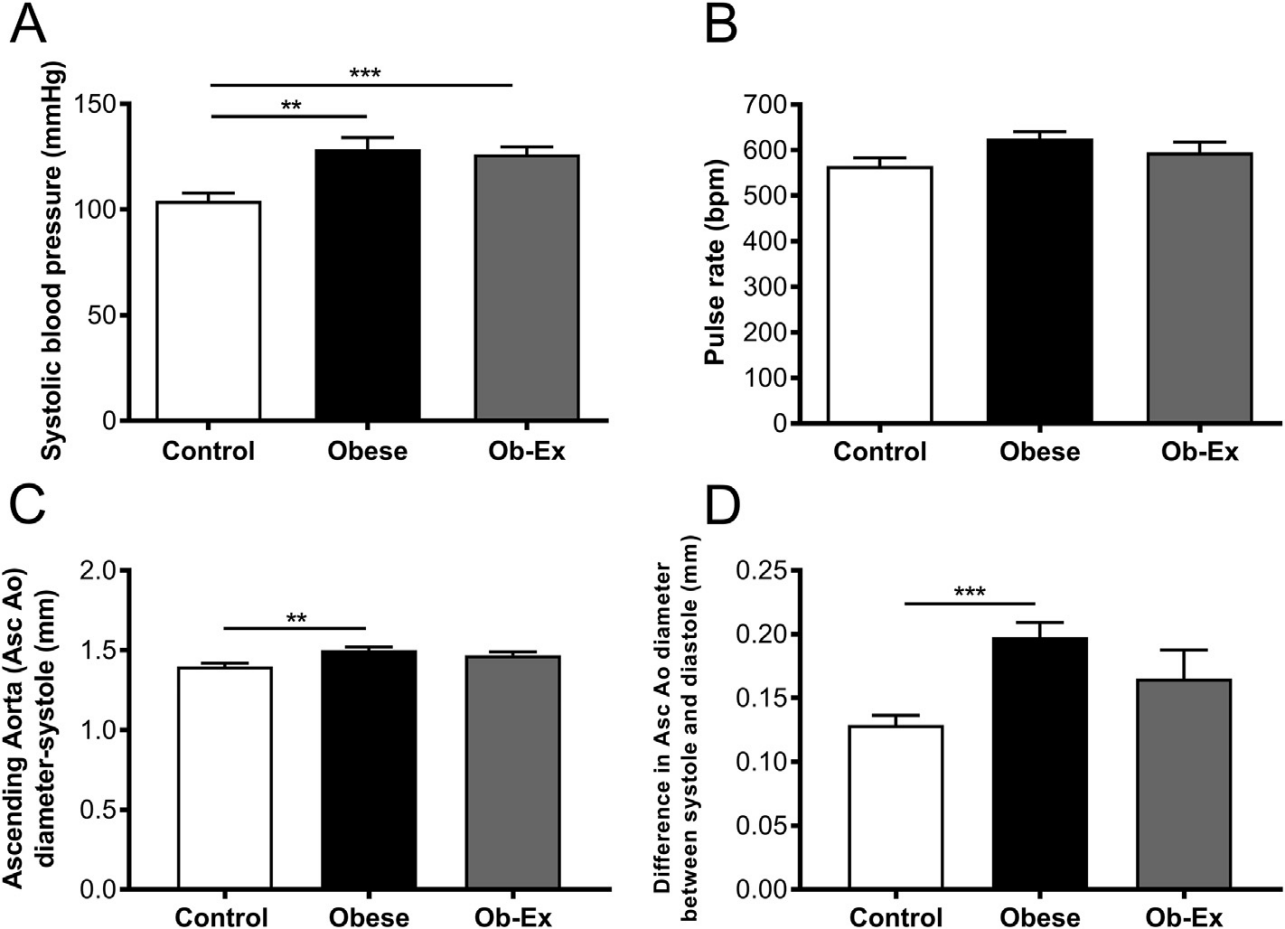
**Offspring blood pressure and aortic diameter**. A) Systolic blood pressure B) Pulse rate (bpm) as measured by tail cuff plethysmography. Control n = 16, Obese n = 14 and Ob-Ex n = 10. C) Ascending aortic (Asc Ao) diameter measured using EKV™ images using echocardiography. Control n = 13, Obese n = 10 and Ob-Ex n = 6. D) Difference in aortic diameter between end-systole and end-diastole. Control n = 10, Obese n = 9 and Ob-Ex n = 4. **p < 0.01 and ***p < 0.001.

Exposure to maternal obesity increased systolic blood pressure. Maternal exercise was unable to prevent this programmed hypertension (Figure 3A). There were no accompanying changes in pulse rate between any of the groups (Figure 3B).

The diameter of the ascending aorta at end-systole was increased in male offspring exposed to maternal obesity and this was not corrected in the Ob-Ex group (Figure 3C). The difference in aortic diameter between end-systole and end-diastole was increased in male offspring exposed to maternal obesity and this was also not corrected by the maternal exercise intervention (Figure 3D).

### 3.5 Echocardiography

**Figure 4:**
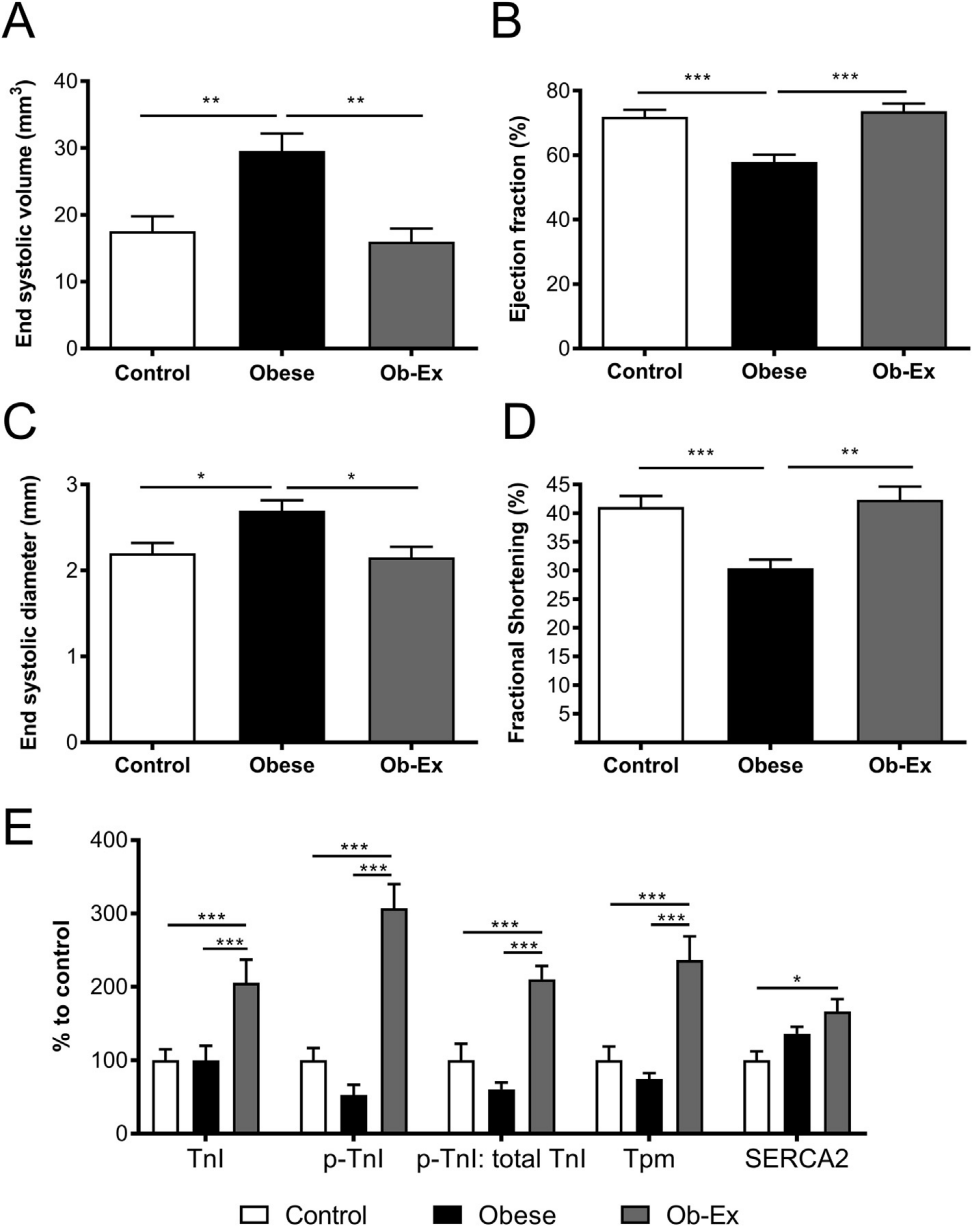
**Offspring cardiac function by *in vivo* echocardiography**. A)-D) LV systolic function and contractility measured by echocardiography. Control n = 16, Obese n = 16 and Ob-Ex n = 7. E) Assessment of proteins important in contractile machinery, blots shown in Supplementary Fig. 2. Control n = 9, Obese n = 8 and Ob-Ex n = 8. *p < 0.05, **p < 0.01 and *** p < 0.001. TnI- total Troponin I, p-TnI- p-Troponin I and Tpm- Tropomyosin.

Left ventricular end-systolic volume (ESV) was elevated in male offspring from obese dams (Figure 4A). This resulted in an accompanying reduction in ejection fraction (Figure 4B). This contributed to a significant overall effect of maternal obesity to reduce cardiac output in the offspring despite no differences in heart rate (Table 3). Furthermore, diameter of the ventricular lumen was increased at the end of systole in offspring of obese mothers suggesting impaired cardiac contractility (Figure 4C). This was confirmed by a reduction in ventricular fractional shortening in the offspring of obese dams (Figure 4D).

**Table 3:** *In vivo* echocardiographic parameters.

	Control	Obese	Ob-Ex	p value
End diastolic volume (μl)	58.9 ± 3.9	68.4 ± 3.0	59.2 ± 3.0	0.097
End diastolic diameter (mm)	3.70 ± 0.10	3.95 ± 0.08	3.72 ± 0.08	0.092
Stroke volume (μl)	41.39 ± 1.9	38.85 ± 1.3	43.26 ± 1.4	0.250
Heart Rate (bpm)	443 ± 12	421 ± 11	446 ± 17	0.291
Cardiac output (ml/min)	18.2 ± 0.8	16.31 ± 0.6	19.37 ± 1.18	**0.046**
IVS; diastole (mm)	0.924 ± 0.04	0.869 ± 0.05	0.957 ± 0.05	0.440
LVID; diastole (mm)	3.65 ± 0.10	3.92 ± 0.07	3.71 ± 0.07	0.061
LVPW; diastole (mm)	0.85 ± 0.04	0.82 ± 0.03	0.89 ± 0.07	0.620
Wall: Lumen ratio; diastole	0.24 ± 0.01	0.21 ± 0.01	0.24 ± 0.02	0.279
IVS; systole (mm)	1.37 ± 0.05	1.25 ± 0.05	1.51 ± 0.09^c^	**0.019**
LVID; systole (mm)	2.32 ± 0.12	2.82 ± 0.10^ac^	2.24 ± 0.14	**0.003**
LVPW; systole (mm)	1.34 ± 0.05	1.18 ± 0.05	1.42 ± 0.11^c^	**0.030**
Wall: Lumen ratio; systole	0.57 ± 0.04	0.43 ± 0.03	0.65 ± 0.08^c^	**0.007**
LV mass (mg)	90.2 ± 3.6	98.0 ± 3.9	97.6 ± 10.0	0.453

LV = Left ventricle, IVS= Interventricular septum, LVID = LV internal diameter and LVPW = LV posterior wall. Control n = 16, Obese n = 16 and Ob-Ex n = 7. *P* values were calculated by one-way ANOVA and shown in **bold** when a significant difference was detected (*p* < 0.05). Letters represent differences between specified groups by Bonferroni post hoc test (p < 0.05) ^a^ Control vs. Obese, ^b^ Control vs. Ob-Ex and ^c^ Obese vs. Ob-Ex.

Offspring from the Ob-Ex group no longer showed an increased left ventricular ESV and end-systolic diameter, and the ejection fraction and fractional shortening was restored to values matching control offspring (Figure 4A–D). Structurally, the diameter of the IVS and LVPW at systole in the intervention group was increased compared to the offspring of non-exercised obese dams. This resulted in an increased systolic wall: lumen width ratio in the offspring of the exercise group. There were no significant differences in the wall or lumen measurements between any groups at the end of diastole (Table 3).

### 3.6 Assessing cardiac contractile machinery proteins

Although maternal obesity did not have any effect on the levels of any of the key cardiac contractile machinery proteins (compared to the control group), levels of Troponin I and Tropomyosin were increased over two-fold in the heart tissue from the Ob-Ex group compared to controls (Figure 4E). Levels of activated p-Troponin I (Ser23/24) were also increased, which reflected an increase in the p-Troponin I: total Troponin I ratio compared to the other two groups. Furthermore, SERCA2, a protein involved in the recycling of calcium, was also increased in the adult male offspring from the Ob-Ex group compared to the other groups (Figure 4E).

## 4 Discussion

The current study addressed the effectiveness of a maternal treadmill exercise intervention during obese pregnancy at preventing programmed cardiovascular dysfunction in male offspring. We found that 20 min of exercise, 5 days a week, 1 week prior to and during pregnancy prevented programmed development of pathologic cardiac hypertrophy and cardiac dysfunction at 8 weeks of age without normalizing maternal body weight. However, despite the clear rescue of the offspring cardiac phenotype in the current study (and metabolic phenotype in previously published studies by us [25] and others [28]), maternal exercise intervention did not prevent hypertension. This highlights divergence of programming pathways that lead to cardiac dysfunction and hypertension. In addition, at a translational level, this provides important evidence that it is not always necessary to alter maternal body weight or fat mass following intervention to improve the offspring phenotype. As many human intervention studies have not observed changes in gestational weight gain in the mother, it offers an important message that exercise without weight loss can still have long-term benefit, and it is therefore critical to study offspring long-term.

### 4.1 Maternal exercise prevented offspring pathologic cardiac hypertrophy and cardiac dysfunction

During cardiac stress such as hemodynamic overload, there is re-expression of fetal genes, which can lead to decompensation and heart failure [29]. Induction of *Nppa* and *Myh7* are highly conserved features of ventricular pathological hypertrophy [30], [31]. *Myh6* (α-MHC, adult isoform) and *Myh7* (β*-*MHC, fetal isoform) are two functionally distinct cardiac isoforms; *Myh7* has a lower sliding velocity therefore an increased ratio of *Myh7*: *Myh6* negatively impacts on cardiac function [32]. Maternal obesity caused the upregulation of *Nppa* and *Myh7* in cardiac tissue of adult male offspring, concurrent with other markers of pathologic cardiac hypertrophy including increased heart weight and cardiomyocyte cell area.

While the development of pathologic cardiac hypertrophy and cardiac dysfunction has been well described in the offspring of obese dams [21], [22], the effects of maternal exercise in obese pregnancy on the cardiovascular health of the offspring has yet to be elucidated. Maternal exercise was beneficial to molecular and histologic markers of pathologic cardiac hypertrophy as well as restoring offspring cardiac function to control levels.

### 4.2 Discordant programming pathways

Consistent with previous studies, offspring of obese dams were hypertensive compared to controls [24], [33]. Despite correcting cardiac dysfunction, maternal exercise intervention failed to prevent offspring hypertension. These findings highlight discordance between the mechanisms through which maternal obesity during pregnancy mediates the programming effects on offspring cardiac dysfunction, compared to those leading to hypertension. Our data suggest that cardiac hypertrophy measured in offspring of obese dams is not a consequence of hypertension but instead programmed through a mechanism modifiable through maternal exercise intervention. The fact that hypertension was not corrected in the offspring exposed to maternal exercise intervention leads us to assess maternal factors not corrected by the intervention as a potential mechanism. The exercise intervention improved maternal insulin sensitivity [25]; therefore, maternal hyperinsulinemia could play a role in mediating offspring cardiac dysfunction but clearly improving insulin sensitivity was insufficient to prevent the increased blood pressure in the offspring. Therefore, a different programming factor is likely mediating the adverse effects on offspring blood pressure. One such factor is maternal leptin, which we [25] and others [28] have shown is not corrected by maternal exercise intervention. It has been shown that inducing experimental hyperleptinemia in neonatal rat pups from postnatal day 9–15 causes increased blood pressure in adulthood [34] and programs long-term renal structural and functional damage through renal sympathetic nerve activation [35]. It has also been suggested that an exaggerated neonatal leptin surge stimulates hypothalamic melanocortin signaling leading to altered sympathetic tone [24]. The lack of effectiveness at the exercise intervention to prevent offspring hypertension, therefore, may be related to the maintained hyperleptinaemia in the exercised obese dams.

In humans persistent hypertension can cause significant enlargement of the aortic root width, compared to age and sex-matched populations of similar body size [36]. We observed a greater increase in aortic width following ventricular contraction, consistent with our functional data that show adult offspring from obese dams experience an increased afterload. The afterload in these offspring was great enough to adversely impact on left ventricular systolic function and myocardial contractility, as ESV was increased, and fractional shortening and ejection fraction were reduced. This suggests that adult offspring of obese mothers cannot maintain cardiac output, a pre-eminent feature of heart failure [37]. Failure to maintain adequate cardiac workload has been shown *ex vivo* in fetal sheep offspring exposed to maternal overnutrition [38].

### 4.3 Mechanisms underlying improved offspring cardiac dysfunction following maternal exercise intervention

The offspring from exercised obese dams showed an improvement in cardiac function despite remaining hypertensive and experiencing increased afterload. Restored ejection fraction, cardiac output and fractional shortening suggest these offspring are overcoming their increase in afterload through compensatory increases in cardiac contractility. Modulation of calcium pathways is critical for the heart to adapt to changing workloads [39]. Key proteins in these pathways were elevated in ventricular tissue from offspring of obese-exercised mothers compared to offspring of both control and non-exercised obese mothers, supporting a role for increased inotropy as a potential driver of their improved cardiac function. Sympathetic activation occurs through β1-adrenergic receptor, which activates Protein Kinase A and the phosphorylation of Troponin I; this increases the calcium sensitivity of Troponin C causing an increased off-rate of calcium, allowing accelerated cardiac relaxation [40]. An up-regulation of proteins in this pathway is indicative of an enhanced sympathetic response. Furthermore, elevated SERCA2 expression would allow calcium to be recycled back to the sarcoplasmic reticulum facilitating efficient future cardiac contractions. Total Troponin I and Tropomyosin in the myocardium were also increased and could further explain the improved cardiac function.

### 4.4 Conclusions

Whilst not all exercise and lifestyle intervention strategies during pregnancy have proved to have immediate benefit to the mother and neonate, our study highlights the importance of exercise in obese pregnancy in reducing long-term cardiovascular risk to the offspring and, therefore, the need for follow up studies across the life course. Just a small improvement in offspring risk could have important implications for the future burden of CVD worldwide. Mechanistically, our study demonstrates that offspring cardiac hypertrophy and dysfunction can be programmed independently of hypertension by adverse intrauterine conditions. Therefore, there is a potential need for combination intervention strategies to tackle the epidemic of obesity in pregnancy, to help reduce all aspects of the future burden of CVD onto the next generation.

## Sources of funding

This work received funding from the British Heart Foundation [PG/13/46/30329, RG/17/12/33167], the European Union's Seventh Framework Programme [FP7/2007-2013, project EarlyNutrition, grant agreement n°289346], MRC Metabolic Diseases Unit award [MC_UU_12012/4] and British Heart Foundation Studentship [(to JB) FS/14/59/31282]. LD is a Sir Henry Wellcome Post-Doctoral Fellow (106026/Z/14/Z).
