# Supplementary material for: Maternal exercise intervention in obese pregnancy improves the cardiovascular health of the adult male offspring
Source: Mol Metab. 2018 Jun 18;16:35–44. doi: 10.1016/j.molmet.2018.06.009 (PMC6157615; doi:10.1016/j.molmet.2018.06.009)
Supplement: Multimedia component 1 [file mmc1.docx]

**Supplementary material**

**Maternal exercise intervention in obese pregnancy improves the cardiovascular health of the adult male offspring**

Jessica H Beeson, Heather L Blackmore PhD, Sarah K Carr PhD, Laura Dearden PhD, Daniella E Duque-Guimarães PhD, Laura C Kusinski PhD, Lucas C Pantaleão PhD, Adele G Pinnock, Catherine E Aiken PhD, Dino A Giussani PhD, Denise S Fernandez Twinn PhD, Susan E Ozanne PhD

**Note:** We would like the supplemental material to be available upon publication.

**Supplementary Table 1: Energy content breakdown of the diets**

|  | *Control* | *Obesogenic* | |
| --- | --- | --- | --- |
|  | *Chow RM1* | *HF pellet* | *Condensed milk* |
| *Metabolisable energy (kJ/g) [kcal/g]* | 13.75  [3.29] | 19.0  [4.54] | 13.63  [3.26] |
| *Energy content (%AFE)* | | | |
| *Fat* | 7.42 | 45 |  |
| *Carbohydrate* | 75.09 | 35 |  |
| *Protein* | 17.49 | 20 |  |

Rodent Maintenance 1 (RM1) and obesogenic (high-fat [HF] pellets, custom-made diet 824053 [SDS]; and condensed milk [Nestlé]) diets were used. Metabolisable energy content is taken from Atwater Fuel Energy (AFE) values given in the dietary information sheets. The Atwater general factor system accounts for the loss of energy from the total available via excretion and secretion. The energy values are 17 kJ/g (4.0 kcal/g) for protein, 37 kJ/g (9.0 kcal/g) for fat and 17 kJ/g (4.0 kcal/g) for carbohydrates.

**Supplementary Table 2: Primer sequences for gene expression of pathologic cardiac hypertrophy markers**

| *Gene name* | *Forward primer (5’ to 3’)* | *Reverse primer (3’ to 5’)* |
| --- | --- | --- |
| *Gapdh* | AGAGTGTTTCCTCGTCCCGT | GCTGGGGAAGTAACTGGAG |
| *Nppa* | ATCGGAGCCTACGAAGATCCA | TTCGGTACCGGAAGCTGTT |
| *Nppb* | AGTCCTTCGGTCTCAAGGCA | AACTTCAGTGCGTTACAGCC |
| *Myh6* | CTGTTCCTCTCTCCGTCCAG | ATTCTGTCACTCAAACTCTGGTTA |
| *Myh7* | GCCAACTATGCTGGAGCTGA | GCAGACACGGTCTGAAAGGA |

**
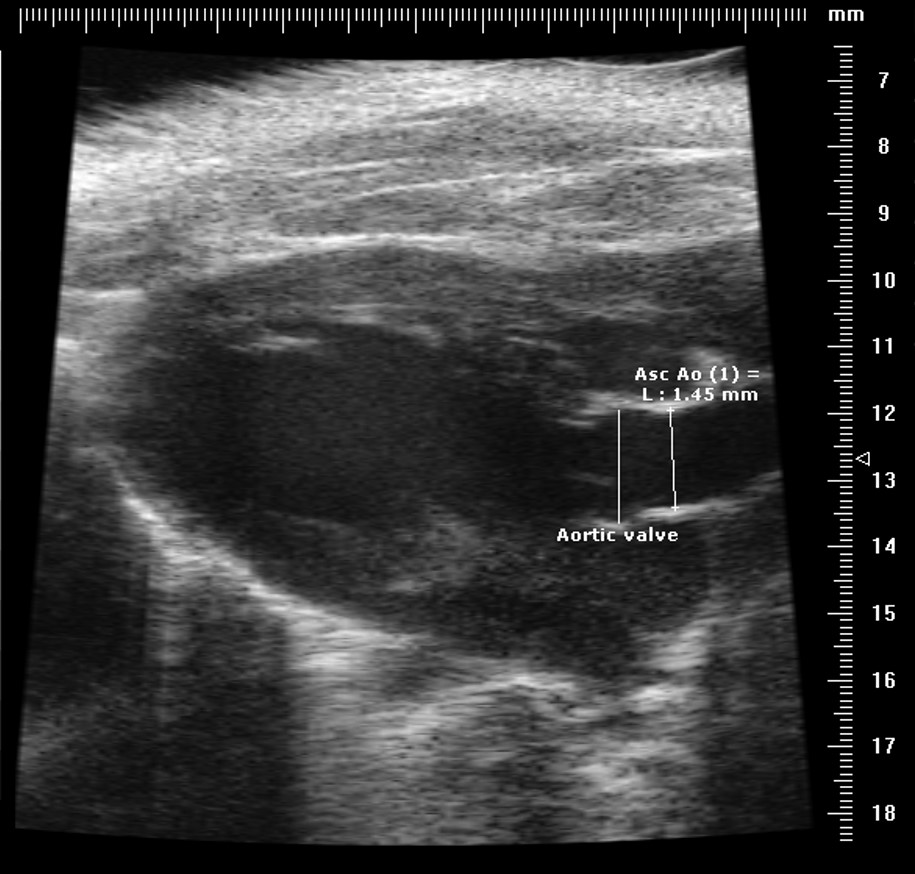
**

**Supplementary Figure 1: EKV^TM^ image of the LV in the parasternal long axis view showing measurements of ascending aorta width.** Ascending aorta width was measured within 1 mm of the aortic valve (labelled on figure) at both end-systole (pictured) and end-diastole (not shown).


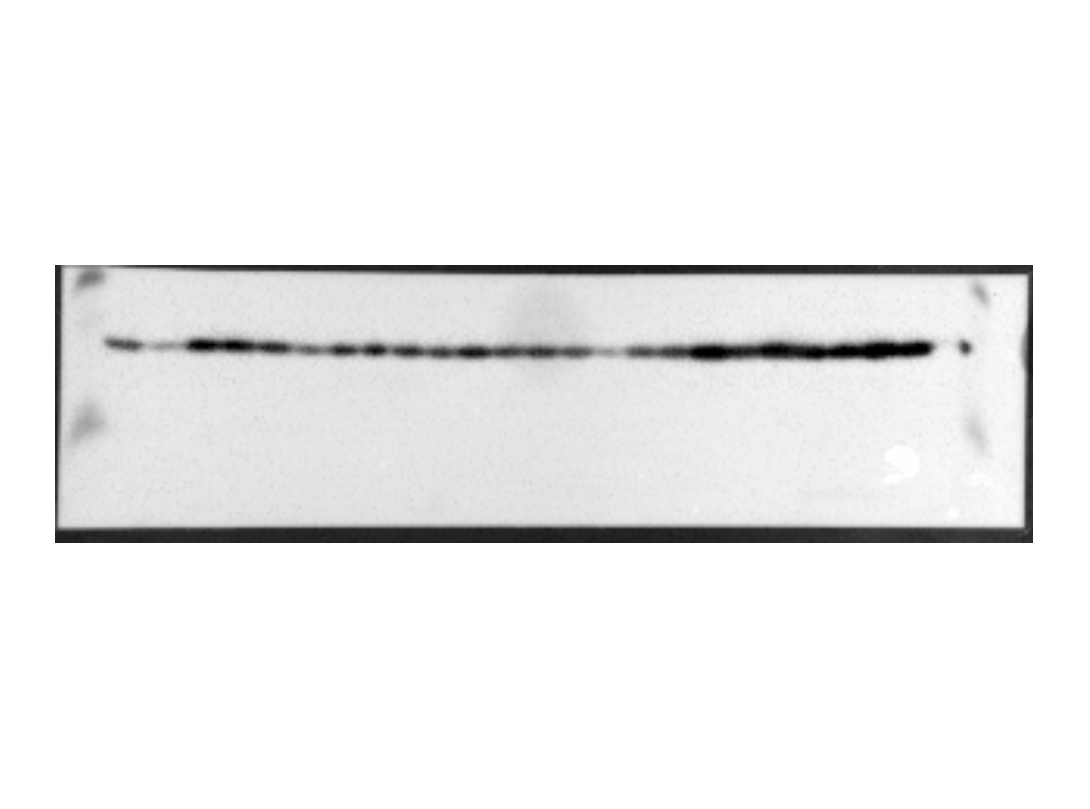


Control

Obese

Ob-Ex

Total Troponin I


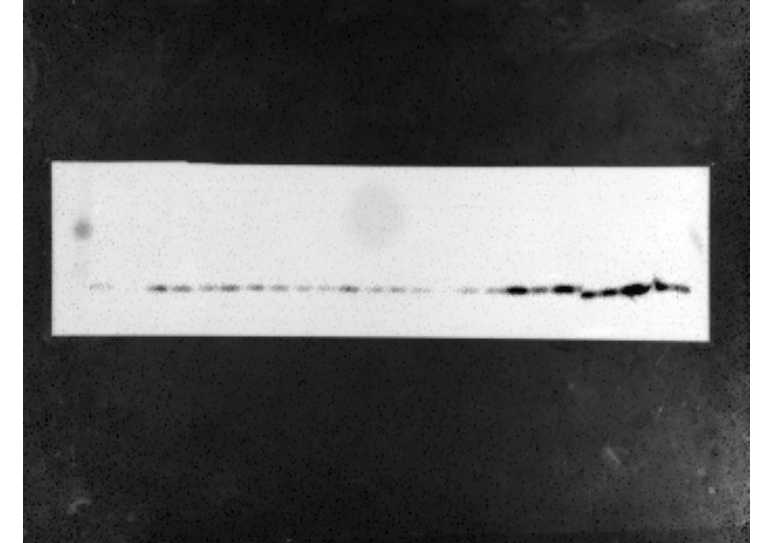


p- Troponin I

Control

Obese

Ob-Ex


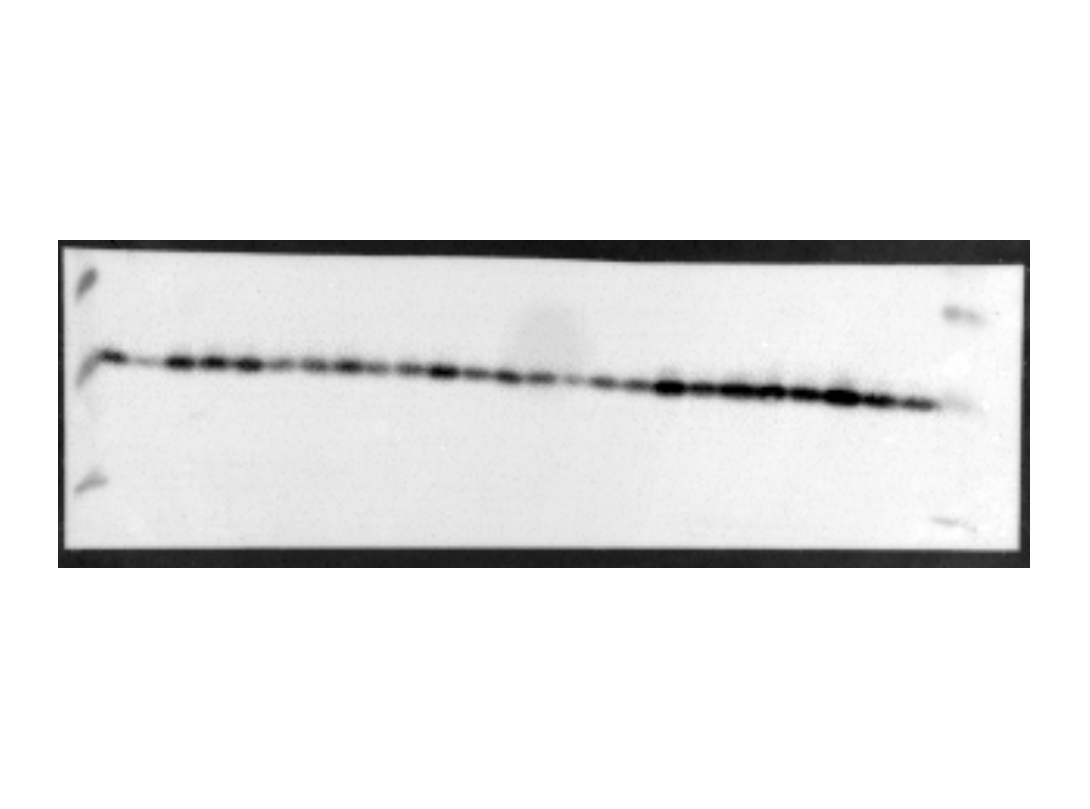


Tropomyosin

Control

Obese

Ob-Ex


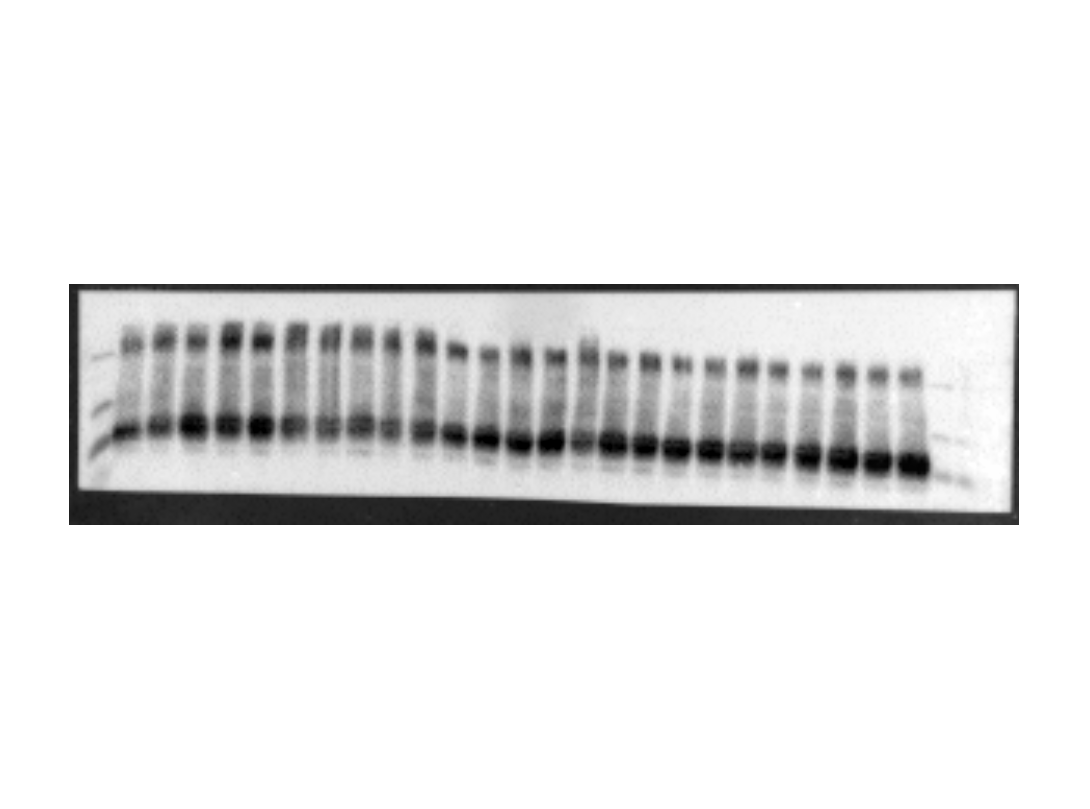


SERCA2

Control

Obese

Ob-Ex

25 kDa

25 kDa

35 kDa

100 Kda

**Supplementrary Figure 2: Western blot images for contractile proteins**
